# Supplementary material for: Fluorescence-detection size-exclusion chromatography utilizing nanobody technology for expression screening of membrane proteins
Source: Commun Biol. 2021 Mar 19;4:366. doi: 10.1038/s42003-021-01891-y (PMC7979870; doi:10.1038/s42003-021-01891-y)
Supplement: Supplementary file 2 — Supplementary Information [file 42003_2021_1891_MOESM2_ESM.pdf]

Supplementary Information

Fluorescence-detection size-exclusion chromatography utilizing nanobody technology for expression screening of membrane proteins

Fei Jin[1], Cheng Shen[1], Yao Wang[1], Mengqi Wang[1], Minxuan Sun[1], Motoyuki Hattori[1].

[1]State Key Laboratory of Genetic Engineering, Shanghai Key Laboratory of Bioactive Small Molecules, Collaborative Innovation Center of Genetics and Development, Department of Physiology and Biophysics, School of Life Sciences, Fudan University, Shanghai 200438, China

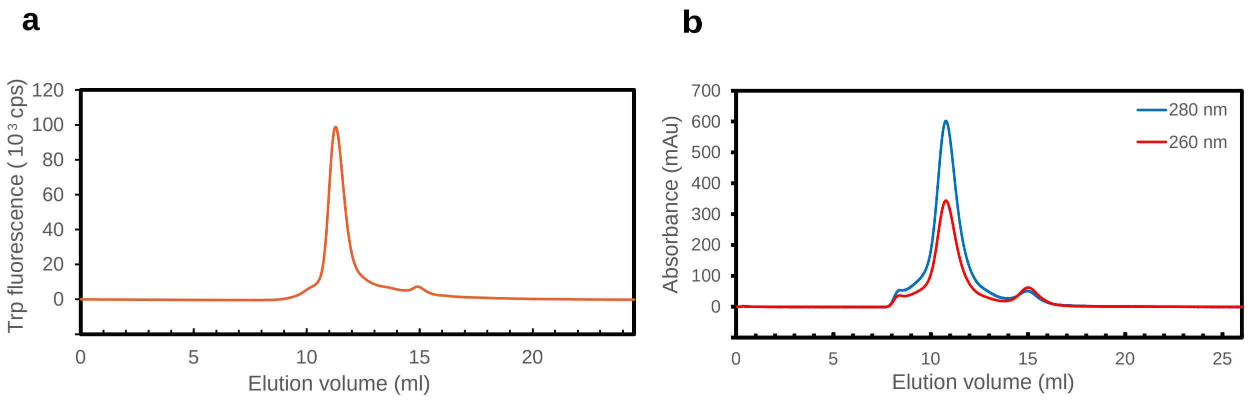

Supplementary Fig. 1 Amphipol reconstitution of OlZAC
(**a**) FSEC trace of NaPol-reconstituted OlZAC on a small scale, as detected by Trp fluorescence. (**b**) Size-exclusion chromatography of NaPol-reconstituted OlZAC, as detected by UV absorbance.

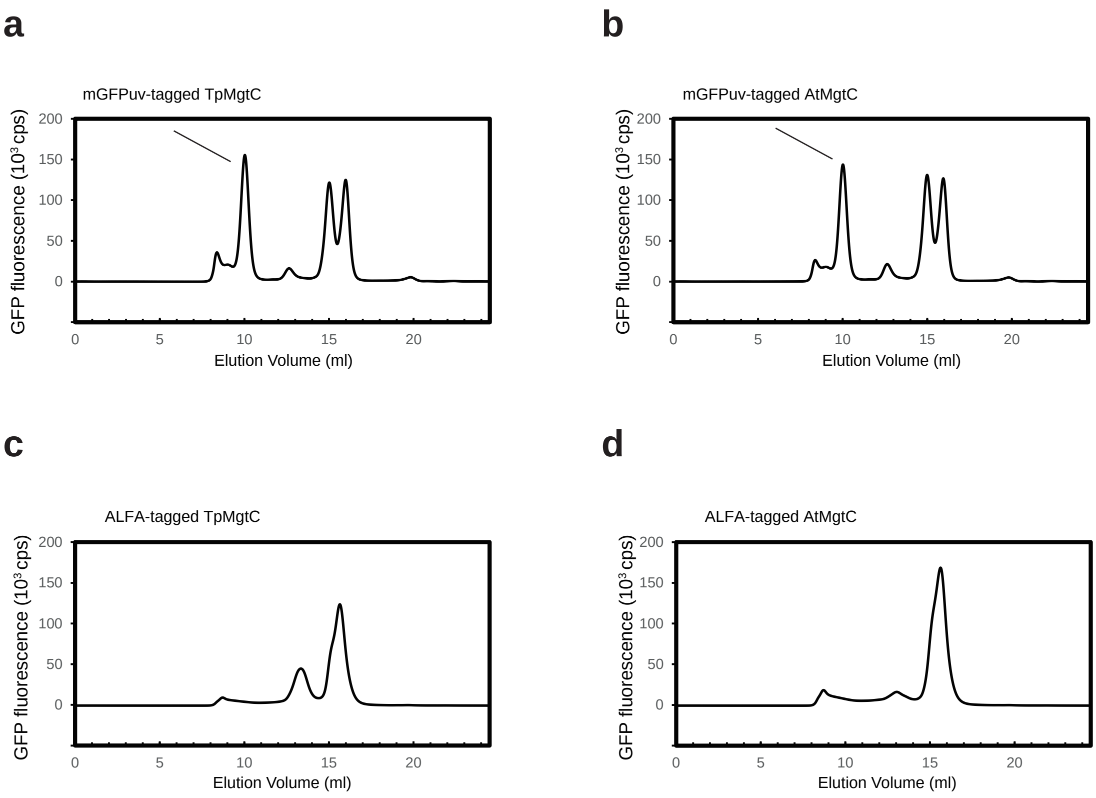

Supplementary Fig. 2 Expression screening of MgtC by GFP-fusion FSEC
(**a, b**) FSEC traces of C-terminally mGFPuv-tagged TpMgtC (Accession Number: WP_038038224.1) and AtMgtC (WP_043965058.1), as detected by mGFPuv fluorescence. (**c, d**) FSEC-Nb traces of ALFA-tagged TpMgtC and AtMgtC with mEGFP-tagged NbALFA, as detected by mEGFP fluorescence.

No	Species	Accession Number	Abbreviation
1	Halobacillus halophilus	WP_014641389.1	HhMgtC
2	Lactococcus lactis	WP_021722089.1	LlMgtC
3	Mycobacterium aromaticivorans	WP_051660484.1	MaMgtC
4	Virgibacillus halodenitrificans	WP_060678220.1	VhMgtC
5	Klebsiella oxytoca	WP_049112851.1	KoMgtC
6	Yersinia ruckeri	WP_004722955.1	YrMgtC
7	Elizabethkingia meningoseptica	WP_019051280.1	EmMgtC
8	Methylophilus sp.	WP_049639727.1	MsMgtC
9	Pedobacter agri	WP_010599226.1	PaMgtC
10	Thermobacillus composti	WP_015253117.1	TcMgtC
11	Thermobispora bispora	WP_013130974.1	TbMgtC
12	Brevibacillus brevis	WP_017246836.1	BbMgtC
13	Vibrio vulnificus	WP_045614446.1	VvMgtC
14	Clostridium acetobutylicum	WP_010966920.1	CaMgtC
15	Xanthobacter autotrophicus	WP_012113199.1	XaMgtC
16	Bacillus cereus	KYQ01271.1	BcMgtC
17	Gracilibacillus boraciitolerans	GAE93792.1	GbMgtC
18	Lactobacillus paracasei	WP_016383475.1	LpMgtC
19	Rubrobacter xylanophilus	WP_011565853.1	RxMgtC
20	Lysinibacillus boronitolerans	WP_016994404.1	LbMgtC
21	Desulfurispora thermophila	WP_018084380.1	DtMgtC
22	Thermanaerothrix daxensis	WP_054522013.1	TdMgtC
23	Thermocrinis albus	WP_012992317.1	TaMgtC
24	Sphaerobacter thermophilus	ACZ39839.1	StMgtC
25	Thermorudis peleae	WP_038038224.1	TpMgtC
26	Rubellimicrobium thermophilum	WP_040645344.1	RtMgtC
27	Quasibacillus thermotolerans	WP_039233714.1	QtMgtC
28	Thermincola ferriacetica	WP_083436703.1	TfMgtC
29	Caldicellulosiruptor naganoensis	WP_045165679.1	CnMgtC
30	Anoxybacillus thermarum	WP_043965058.1	AtMgtC
31	Desulfofundulus	WP_027355452.1	DeMgtC
32	Thermodesulfobium narugense	WP_013756617.1	TnMgtC
33	Serratia	WP_006319255.1	SeMgtC
34	Bacillus mannanilyticus	WP_025026422.1	BmMgtC
35	Alkalihalobacillus akibai	WP_035663104.1	AaMgtC
36	Hungateiclostridium thermocellum	WP_003513884.1	HtMgtC
37	Stenotrophomonas pictorum	WP_054658310.1	SpMgtC
38	Prevotella maculosa	WP_019967744.1	PmMgtC
39	Prevotella salivae	EFV04256.1	PsMgtC

40	Prevotella veroralis	WP_018911053.1	PvMgtC
41	Prevotella sp.	WP_177216065.1	PspMgtC
42	Peptococcaceae bacterium	KJS46311.1	PbMgtC
43	Sphingobacteriales bacterium	OJV97509.1	SbMgtC
44	Yersinia pestis	WP_015683614.1	YpMgtC
45	Natronincola peptidivorans	WP_090446121.1	NpMgtC
46	Myxococcus hansupus	WP_021781415.1	MhMgtC
47	Obesumbacterium proteus	WP_046459523.1	OpMgtC
48	Limihaloglobus sulfuriphilus	WP_146682549.1	LsMgtC
49	Lactobacillus kefiri	WP_054769137	LkMgtC
50	Dictyoglomus sp.	PMQ01502.1	DspMgtC
51	Bacillus sp.	WP_094032362.1	BspMgtC
52	Sphingobacterium detergens	WP_120259343.1	SdMgtC
53	Chryseobacterium culicis	WP_105683769	CcMgtC
54	Lactobacillaceae	WP_021357857.1	LaMgtC
55	Bacteroides eggerthii	WP_118363478.1	BeMgtC
56	Acidaminococcus	WP_016459447.1	AcMgtC
57	Tissierella sp. P1	WP_094904138.1	TspMgtC
58	Mucilaginibacter sp.	WP_067187481.1	MspMgtC
59	Erwinia typographi	WP_034897147.1	EtMgtC
60	Clostridium tepidiprofundi	WP_066821746.1	CtMgtC
61	Arthrobacter sp.	WP_155850019.1	AsMgtC
62	Chitinophagaceae bacterium	WP_157444983.1	CbMgtC
63	Risungbinella massiliensis	WP_044641850.1	RmMgtC
64	Deltaproteobacteria bacterium	OGR23206.1	DbMgtC
65	Carnobacterium iners	WP_085559504.1	CiMgtC
66	Lactobacillus farraginis	KRM01365.1	LfMgtC
67	Thermosyntropha lipolytica	WP_073089568.1	TlMgtC
68	Acidobacteria bacterium	PYX87356.1	AbMgtC

Supplementary Table 1 MgtC orthologs for GFP fusion-based FSEC screening
