# Supplementary material for: Fluorescence-detection size-exclusion chromatography utilizing nanobody technology for expression screening of membrane proteins
Source: Commun Biol. 2021 Mar 19;4:366. doi: 10.1038/s42003-021-01891-y (PMC7979870; doi:10.1038/s42003-021-01891-y)
Supplement: Supplementary file 3 — Description of Additional Supplementary Files [file 42003_2021_1891_MOESM3_ESM.pdf]

## Description of Additional Supplementary Files

**File name:** Supplementary Data

**Description:** All source data underlying the graphs and charts.
